# Risk factors for non-curative resection of early gastric neoplasms with endoscopic submucosal dissection: Analysis of 1,123 lesions

**DOI:** 10.3892/etm.2015.2265

**Published:** 2015-02-05

**Authors:** TATSUYA TOYOKAWA, TOMOKI INABA, SHIZUMA OMOTE, AKIKO OKAMOTO, RIKA MIYASAKA, KAZUO WATANABE, KOICHI IZUMIKAWA, ISAO FUJITA, JOICHIRO HORII, SHIGENAO ISHIKAWA, TAMIYA MORIKAWA, TAKAKO MURAKAMI, JUN TOMODA

**Affiliations:** 1 Department of Gastroenterology, National Hospital Organization, Fukuyama Medical Center, Fukuyama, Hiroshima, Japan; 2 Department of Gastroenterology, Kagawa Prefectural Central Hospital, Takamatsu, Kagawa, Japan

**Keywords:** endoscopic submucosal dissection, early gastric neoplasms, non-curative resection, risk factors

## Abstract

Although the frequency of residual disease and recurrence following endoscopic submucosal dissection (ESD) has markedly decreased, a few cases of residual disease and recurrence following ESD are still observed. The aims of the present study were to clarify the causes of non-curative resection and to investigate the risk factors. A total of 1,123 early gastric neoplasm lesions treated by ESD were investigated. Non-curative resection was defined as histological positivity of the resected margins, vascular invasion or failure of *en bloc* resection. Cases of non-curative resection were classified as being caused by one of three reasons: Inadequate technique, pre-procedural misdiagnosis or problems in the histological diagnosis. Following classification, the cases of non-curative and curative resection were compared based on a range of patient characteristics: Procedure time, and size, type and location of the lesions. The frequency of non-curative resection was 16% (182 lesions). Non-curative resection occurred due to inadequate technique in 59 cases, pre-procedural misdiagnosis in 88 cases and problems in the histological diagnosis in 35 cases. Multivariate analysis revealed that a large lesion size, long procedure time and inexperienced endoscopist were associated with a significantly higher risk of non-curative resection due to an inadequate technique. Furthermore, it was found that lesions located in the upper area of the stomach and cancer with submucosal invasion were associated with a significantly higher risk of non-curative resection due to pre-procedural misdiagnosis. In conclusion, the present study has shown that the major reasons for non-curative resection are an inadequate technique and pre-procedural misdiagnosis. The risk factors for these problems have been clarified.

## Introduction

Endoscopic submucosal dissection (ESD) facilitates *en bloc* resection and is therefore considered to be a useful procedure for the treatment of early gastric neoplasms (1,2). Through *en bloc* resection, early gastric neoplasms can be completely removed, following which the specimens can be accurately evaluated by histological examination (1,3). In Japan, ESD has been established as a standard treatment for early gastric neoplasms; the popularity of the procedure has also been enhanced on a global scale (4–7).

The frequency of residual disease and recurrence following ESD has markedly decreased compared with that following conventional endoscopic mucosal resection (EMR), as the ESD procedure facilitates *en bloc* resection (1,2,8,9); however, a few cases of residual disease and recurrence following non-curative resection by ESD are still observed (10–12). There are various reasons for non-curative resection, including a failure to perform *en bloc* resection due to an inadequate technique or a pre-procedural misdiagnosis of the margin or depth of the lesion. ESD is an excellent procedure for the treatment of early gastric neoplasms; however, it requires advanced endoscopic skills and has a higher incidence of complications, such as perforation and bleeding, compared with conventional EMR methods (1,2,4). It is additionally difficult to perform the ESD procedure for lesions at certain locations and of large sizes. Thus, in certain cases, *en bloc* resection cannot be performed as expected. The prediction of neoplasm margins or depths can be difficult at times due to the fact that the background of the gastric mucosa is affected by acute or chronic inflammation (8). This can result in incorrect prediction of the margin or depth of the lesions, despite of the use of chromoendoscopy with indigocarmine dye or magnifying endoscopy with narrow-band imaging (NBI) (8,13). It is therefore important to clarify the causes of such errors and to investigate the various risk factors for non-curative resection.

## Materials and methods

### Patients and ESD procedure

A total of 967 patients (1,123 lesions) diagnosed with early gastric neoplasms were recruited for this study. The patients had undergone ESD at Fukuyama Medical Center (Fukuyama, Japan), Mitoyo General Hospital (Kanonji, Japan) or Kagawa Prefectural Hospital (Takamatsu, Japan) between May 2003 and August 2010. The cases were all diagnosed as gastric adenoma or adenocarcinoma. Written informed consent was obtained from all of the patients. ESD was performed as described previously (1,2,14). The knives used for the ESD were the insulation-tip diathermic knife and the flex knife. Seven endoscopists performed the ESD procedures in this study.

### Ethics statement

Approval for this study was obtained from the Institutional Review Board of National Hospital Organization, Fukuyama Medical Center. Reporting of the study conforms to the Strengthening the Reporting of Observational Studies in Epidemiology (STROBE) statement, with references to STROBE and the broader Enhancing the Quality and Transparency of Health Research guidelines.

### Classification of non-curative resection

The indication for ESD in this study was gastric adenoma or intramucosal gastric cancer with predominantly well or moderately differentiated adenocarcinoma, regardless of lesion size. In this study non-curative resection was defined as histological positivity of the resected tissue margins, evidence for a predominantly undifferentiated type, infiltration of veins or lymphatic vessels or submucosal invasion by histological examination of the resected specimens, or procedural failure of *en bloc* resection. Cases of non-curative resection were classified as being due to an inadequate technique, pre-procedural misdiagnosis or problems in the histological diagnosis. Inadequate technique indicated that dissection of the submucosa was incomplete due to a large lesion size, a lesion combined with an ulcer scar, tumor location, complications, poor patient condition or as a result of the resection being performed in a piecemeal approach due to difficulties in performing *en bloc* resection. Pre-procedural misdiagnosis indicated that the cancer cells had spread beyond the marking dots and the resected tissue margins had scored histologically positive on the resected specimens or that cancer cells had infiltrated the submucosal layer of the resected specimens. Problems in the histological diagnosis indicated that lymphatic or venous involvement had been observed on the resected specimens or that an undifferentiated type of adenocarcinoma was detected on the resected specimens.

### Comparison of non-curative and curative resections

To compare the non-curative and curative resections, an analysis was performed on the basis of the following patient characteristics: Length of procedure, and size, type and location of lesions. Comparisons were made with regard to the occurrence of an inadequate technique or pre-procedural misdiagnosis.

### Statistical analysis

Statistical analyses were performed using logistic regression analysis for univariate and multivariate analyses. All statistical analyses were performed using SPPS version 18.0 statistical software (SPSS Inc., Chicago, IL, USA). P<0.05 was considered to indicate a statistically significant difference.

## Results

### Reasons for non-curative resection

The frequency of non-curative resection was 16% (182 lesions). The reasons for non-curative resection were as follows: Inadequate technique, 59 lesions (32%); pre-procedural misdiagnosis, 88 lesions (48%); and problems in the histological diagnosis, 35 lesions (19%) (Table I).

### Inadequate technique

Univariate analysis indicated that the risk factors for non-curative resection due to inadequate technique were a large lesion size, a lesion complicated with an ulcer scar, a long procedure time and inexperienced endoscopists (Table II). Multivariate analysis revealed that a large lesion size [odds ratio (OR), 1.05; 95% confidence interval (CI), 1.03–1.07] and long procedure time (OR, 1.01; 95% CI, 1.00–1.01) were associated with a significantly higher risk of non-curative resection due to an inadequate technique (Table III). Inexperienced endoscopists were also associated with a significantly higher risk of non-curative resection (OR, 1.63; 95% CI, 1.18–2.26; P=0.0034) (Table III).

### Pre-procedural misdiagnosis

Univariate analysis indicated that the risk factors for non-curative resection due to pre-procedural misdiagnosis included lesions located in the upper area of the stomach, a large lesion size, cancer with submucosal invasion and inexperienced endoscopists (Table IV). Multivariate analysis revealed that lesions located in the upper area of the stomach (OR, 1.74; 95% CI, 1.02–2.97) and cancer with submucosal invasion (OR, 24.4; 95% CI, 13.9–41.7) were associated with a significantly higher risk of non-curative resection due to pre-procedural misdiagnosis (Table V).

## Discussion

ESD has been established as the standard treatment for early gastric neoplasms in Japan (1,2). ESD facilitates *en bloc* resection using newly developed endoscopic knives for large lesions (15,16). ESD is an improved procedure, as it can reduce the incidence of local recurrence, which is not a feature of conventional EMR (9), and the *en bloc*-resected specimens can be accurately evaluated by histological examination (1,3). Specimen recovery by *en bloc* resection provides accurate pathological information on the tumor depth, size and lymphovascular infiltration, as well as indications of whether the resected tissue margins are cancer-free (1,3,8). Residual or recurrent disease rarely occurs in cases treated through ESD methods (17,18). Cases of residual or recurrent disease can be predicted by pathological information obtained from the resected specimen. In the present study, the causes of and risk factors for non-curative resection were investigated.

The indications for the endoscopic treatment of gastric cancer have been controversial. In 2010, the Japanese gastric cancer treatment guidelines (version 3) were published (19); however, we experienced the case of lymph node metastasis with undifferentiated gastric adenocarcinoma that was intramucosal cancer with a diameter of 13 mm. In the present study, a predominantly undifferentiated type, infiltration of veins or lymphatic vessels or submucosal invasion by histological examination of the resected specimens were defined as a non-curative resection.

Each risk factor for non-curative resection (inadequate technique, pre-procedural misdiagnosis and problems in histological diagnosis) was independently investigated in this study. The techniques and skills required for diagnostics and those required for conducting the procedure are different. Accordingly, the risk factors for inadequate technique and pre-procedural misdiagnosis were found to differ in this study. Despite this, the diagnostic and surgical procedures share certain similarities. In this study, lesions located in the upper area of the stomach were a risk factor for non-curative resection due to pre-procedural misdiagnosis; however, we have previously demonstrated that this lesion location is also a risk factor for perforation (4). In this study, large lesion size was a risk factor for non-curative resection due to inadequate technique; however, Kakushima *et al* (8) showed that it was also a risk factor for lateral margin positive resection due to misdiagnosis of the tumor extent.

In this study, approximately half of all lesions of non-curative resection were a result of pre-procedural misdiagnosis. In these cases, 57 lesions were misdiagnosed at the lateral margin, 34 were misdiagnosed at the vertical margin and three cases were misdiagnosed at both margins. Lesions located in the upper area of the stomach and cancer with submucosal invasion were associated with a significantly higher risk of non-curative resection due to pre-procedural misdiagnosis. It is logical to conclude that cancer with submucosal invasion was a risk factor; however, why lesions located in the upper area of the stomach were a risk factor remains unclear. We speculated that it was more difficult to make detailed observations in the upper area of the stomach compared with other areas.

New devices have been developed to improve the ESD technique (15,16); however, although these new devices have improved the safety of the ESD procedure, the rate of *en bloc* resection has not improved immediately (15,16). In this study, several devices were used by different endoscopists, and the effect of using different devices was not investigated. Since several devices were sometimes used in one ESD procedure, the effect of the different devices was difficult to investigate.

An inadequate technique accounted for one-third of all non-curative lesion resections in this study. In the univariate analysis, the risk factors were a large lesion size, a lesion complicated with an ulcer scar, a long procedure time and inexperienced endoscopists. It is a logical conclusion that a lesion complicated with an ulcer scar is a risk factor, as it is difficult to dissect the submucosa in the presence of the complication of an ulcer scar (17,20). We propose that long procedure time may be a risk factor for non-curative resection due to the fact that a long procedure time was required to resect difficult lesions.

Various modalities have been used for the pre-procedural diagnosis of lesion extent (13,21). NBI is a useful method for pre-procedural diagnosis (13). Chromoendoscopy with an acetic acid-indigocarmine mixture (AIM) reportedly improves pre-procedural diagnosis by delineating the margin of the lesions (21). In the present study, the pre-procedural diagnosis of the lesion extent was mainly made by normal white-light endoscopy and chromoendoscopy. NBI and AIM were used for certain lesions, but with a smaller patient cohort; therefore, investigations into whether these factors contributed to pre-procedural misdiagnosis were not performed. In the future, these methods should be studied and tested in order to determine whether they are risk factors for non-curative resection due to pre-procedural misdiagnosis.

The diagnosis of tumor depth is difficult, even with modern techniques (18,22); however, although numerous methods have been attempted for the pre-procedural diagnosis of lesion depth, no method has been conspicuous without endoscopic ultrasound (EUS) (23). In the present study EUS was used for the pre-procedural diagnosis of lesion depth; however, not all lesions were examined using this method. Even EUS lacks sufficient accuracy; therefore, new methods are likely to be developed in the future.

In conclusion, it was demonstrated in the present study that non-curative resection occurred at low frequencies following ESD for early gastric neoplasms, and the reasons for non-curative resection were mainly attributable to an inadequate technique and incorrect pre-procedural misdiagnosis. In addition, the risk factors for non-curative resection were clarified. These results highlight the necessity of conducting ESD procedures more accurately in cases of early gastric neoplasms.

## Figures and Tables

**Table I:** Reasons for non-curative resection.

Reason	Lesions, n (%)
Inadequate technique	59 (32)
Pre-procedural misdiagnosis	88 (48)
Problems in histological diagnosis	35 (19)

n=182.

**Table II:** Risk factors for non-curative resection due to inadequate technique (univariate analysis).

Risk factor	Non-curative resection	Curative resection	P-value
Number of lesions	59	941	
Number of patients	59	908	
Age in years, median (range)	70 (49–88)	72 (26–95)	NS
Males:Females, n:n	49:10	674:267	0.061
Underlying diseases, n (%)			
Hypertension	20 (34)	388 (41)	NS
Diabetes mellitus	10 (17)	126 (13)	NS
Hyperlipidemia	8 (14)	119 (13)	NS
Heart disease	12 (20)	148 (16)	NS
Cerebrovascular disease	8 (14)	69 (7.3)	0.074
Chronic renal failure	2 (3.4)	17 (1.8)	NS
Antiplatelet agent or anticoagulant use, n (%)	12 (20)	156 (17)	NS
Location, n (%)			NS
Upper	17 (29)	187 (20)	
Middle	24 (41)	377 (40)	
Lower	16 (27)	374 (40)	
Anastomosis	2 (3.4)	3 (0.32)	
Size of lesion in mm, mean (range)	21.8 (5–50)	18.3 (1–85)	<0.0001
Type, n (%)			NS
Elevated	3 (5.1)	17 (1.8)	
Surface elevated	28 (47)	457 (49)	
Surface flat	0 (0.0)	23 (2.4)	
Surface depressed	28 (47)	444 (47)	
Combined ulcer or ulcer scar, n (%)	12 (20)	61 (6.5)	0.0002
Disease, n (%)			NS
Intramucosal gastric cancer	49 (83)	648 (69)	
Gastric cancer with submucosal invasion	4 (6.8)	55 (5.8)	
Gastric adenoma	6 (10)	238 (25)	
Procedure time in min, mean (range)	149 (10–590)	95 (9–640)	<0.0001
Experience of endoscopist, n (%)			0.0001
≤50 cases	34 (58)	324 (34)	
>50 and ≤100 cases	11 (19)	177 (19)	
>100 cases	14 (24)	440 (47)	
Institution, n (%)			NS
A	14 (24)	176 (19)	
B	20 (34)	327 (35)	
C	25 (42)	438 (47)	

NS, not significant; A, Mitoyo General Hospital; B, Fukuyama Medical Center; C, Kagawa Prefectural Central Hospital.

**Table III:** Risk factors for non-curative resection due to inadequate technique (multivariate analysis).

Variable	Odds ratio (95% confidence interval)	P-value
Male gender	1.63 (0.79–3.33)	0.1900
Presence of cerebrovascular disease	1.70 (0.74–3.94)	0.2100
Size of lesion in mm	1.05 (1.03–1.07)	<0.0001
Presence of combined ulcer or ulcer scar	1.72 (0.77–3.85)	0.1900
Procedure time in min	1.01 (1.00–1.01)	0.0005
Endoscopist with <50 cases experience	1.63 (1.18–2.26)	0.0034

**Table IV:** Risk factors for non-curative resection due to pre-procedural misdiagnosis (univariate analysis).

Risk factor	Non-curative resection	Curative resection	P-value
Number of lesions	88	941	
Number of patients	88	908	
Age in years, median (range)	72 (43–87)	72 (26–95)	NS
Males:Females, n:n	66:22	674:267	NS
Underlying diseases, n (%)			
Hypertension	34 (39)	388 (41)	NS
Diabetes mellitus	15 (17)	126 (13)	NS
Hyperlipidemia	8 (9.1)	119 (13)	NS
Heart disease	8 (9.1)	148 (16)	0.11
Cerebrovascular disease	5 (5.7)	69 (7.3)	NS
Chronic renal failure	3 (3.4)	17 (1.8)	NS
Antiplatelet agent or anticoagulant use, n (%)	14 (16)	156 (17)	NS
Location, n (%)			0.0008
Upper	32 (36)	187 (20)	
Middle	33 (38)	377 (40)	
Lower	22 (25)	374 (40)	
Anastomosis	1 (1.1)	3 (0.32)	
Size of lesion in mm, mean (range)	24.6 (5–80)	18.3 (1–85)	<0.0001
Type, n (%)			NS
Elevated	2 (2.3)	17 (1.8)	
Surface elevated	37 (42)	457 (49)	
Surface flat	2 (2.3)	23 (2.4)	
Surface depressed	47 (53)	444 (47)	
Combined ulcer or ulcer scar, n (%)	8 (9.1)	61 (6.5)	
Disease, n (%)			<0.0001
Intramucosal gastric cancer	28 (32)	648 (69)	
Gastric cancer with submucosal invasion	55 (63)	55 (5.8)	
Gastric adenoma	5 (5.7)	238 (25)	
Experience of endoscopist, n (%)			0.024
≤50 cases	25 (28)	324 (34)	
>50 and ≤100	10 (11)	177 (19)	
>100 cases	53 (60)	440 (47)	
Institution, n (%)			NS
A	22 (25)	176 (19)	
B	26 (30)	327 (35)	
C	24 (45)	438 (47)	

NS, not significant; A, Mitoyo General Hospital; B, Fukuyama Medical Center; C, Kagawa Prefectural Central Hospital.

**Table V:** Risk factors for non-curative resection due to pre-procedural misdiagnosis (multivariate analysis).

Variable	Odds ratio (95% CI)	P-value
Presence of heart disease	1.62 (0.69–3.82)	0.27
Upper location	1.74 (1.02–2.97)	0.042
Size of lesion in mm	1.03 (0.99–1.04)	0.051
Disease, gastric cancer with submucosal invasion	24.4 (13.9–41.7)	<0.0001
Endoscopist with <50 cases experience	1.03 (0.76–1.39)	0.87

95% CI, 95% confidence interval.
